# BRET-Based Biosensors to Measure Agonist Efficacies in Histamine H_1_ Receptor-Mediated G Protein Activation, Signaling and Interactions with GRKs and β-Arrestins

**DOI:** 10.3390/ijms23063184

**Published:** 2022-03-16

**Authors:** Eléonore W. E. Verweij, Reggie Bosma, Meichun Gao, Jelle van den Bor, Betty Al Araaj, Sabrina M. de Munnik, Xiaoyuan Ma, Rob Leurs, Henry F. Vischer

**Affiliations:** Division of Medicinal Chemistry, Faculty of Science, Amsterdam Institute of Molecular and Life Sciences, Vrije Universiteit Amsterdam, 1081 HZ Amsterdam, The Netherlands; noortje.verweij@xs4all.nl (E.W.E.V.); r.bosma@vu.nl (R.B.); m.c.gao@vu.nl (M.G.); j.vanden.bor@vu.nl (J.v.d.B.); bettyalaraaj@hotmail.com (B.A.A.); sabrinademunnik@hotmail.com (S.M.d.M.); x.ma@vu.nl (X.M.); r.leurs@vu.nl (R.L.)

**Keywords:** histamine, H_1_R, GPCR, G protein, β-arrestin, GPCR kinase, BRET assay, biased signaling

## Abstract

The histamine H_1_ receptor (H_1_R) is a G protein-coupled receptor (GPCR) and plays a key role in allergic reactions upon activation by histamine which is locally released from mast cells and basophils. Consequently, H_1_R is a well-established therapeutic target for antihistamines that relieve allergy symptoms. H_1_R signals via heterotrimeric G_q_ proteins and is phosphorylated by GPCR kinase (GRK) subtypes 2, 5, and 6, consequently facilitating the subsequent recruitment of β-arrestin1 and/or 2. Stimulation of a GPCR with structurally different agonists can result in preferential engagement of one or more of these intracellular signaling molecules. To evaluate this so-called biased agonism for H_1_R, bioluminescence resonance energy transfer (BRET)-based biosensors were applied to measure H_1_R signaling through heterotrimeric G_q_ proteins, second messengers (inositol 1,4,5-triphosphate and Ca^2+^), and receptor-protein interactions (GRKs and β-arrestins) in response to histamine, 2-phenylhistamines, and histaprodifens in a similar cellular background. Although differences in efficacy were observed for these agonists between some functional readouts as compared to reference agonist histamine, subsequent data analysis using an operational model of agonism revealed only signaling bias of the agonist Br-phHA-HA in recruiting β-arrestin2 to H_1_R over G_q_ biosensor activation.

## 1. Introduction

The histamine H_1_ receptor (H_1_R) is a membrane-associated G protein-coupled receptor (GPCR) that is ubiquitously expressed on vascular endothelial, smooth muscle, immune, and brain cells and involved in, for example, acute allergic reactions, uterine contraction in preterm labor, and awakening in response to the biogenic amine histamine [1,2]. Since the 1940s, H_1_R became a blockbuster drug target for the treatment of allergic responses by two generations of antihistamines, with the first-generation of these antagonists also being sedative and reducing motion sickness by crossing the blood-brain barrier and acting on H_1_R in the brain [3]. H_1_R signals primarily through heterotrimeric G_q/11_ proteins to activate the phospholipase C signaling cascade resulting in the conversion of phosphatidylinositol 4,5-bisphosphate into inositol 1,4,5-triphosphate (InsP_3_) and 1,2-diacylglycerol. These so-called second messengers, in turn, increase intracellular Ca^2+^ levels and protein kinase C activity, resulting in various downstream cellular responses [2]. GPCR-mediated G protein activation is attenuated by phosphorylation of the receptor at intracellular serine and/or threonine residues, resulting in the recruitment of β-arrestin1 and/or 2 that sterically hinder further G protein coupling and facilitate receptor internalization [4,5]. Indeed, H_1_R recruits both β-arrestin1 and 2 in response to histamine stimulation as revealed by bioluminescence resonance energy transfer (BRET) and split-luciferase complementation experiments to detect protein-protein interactions [6,7,8,9]. However, H_1_R desensitization is only mediated by β-arrestin2 in myometrial smooth muscle cells as revealed by knockdown of β-arrestin1 and/or 2 by siRNA [10]. G protein-coupled receptor kinases (GRK) are critically involved in the phosphorylation of GPCRs to drive β-arrestin1/2 recruitment and activity [4]. Indeed, overexpression of GRK5, GRK6, or in particular GRK2, increased the phosphorylation of H_1_R in human embryonic kidney (HEK)293T upon histamine stimulation [11]. Moreover, overexpression of GRK2 abolished H_1_R-induced InsP_3_ production, which required both its catalytic kinase and regulator of G protein signaling (RGS) domains, whereas GRK5 and GRK6 had limited effect [11,12]. In line, knockdown of these individual GRK subtypes by siRNA confirmed that only GRK2 plays a role in H_1_R desensitization in HEK293 and myometrial smooth muscle cells upon stimulation with histamine [11,12].

GPCR ligands can induce activity bias towards certain intracellular effectors at the expense of others, by stabilizing distinct active receptor conformations [13,14]. For example, the selective engagement of GRK subtypes in response to different agonists can change the phosphorylation pattern at the intracellular loops and C-tail of GPCRs and consequently dictate subsequent β-arrestin1/2-mediated activities [15,16,17,18,19]. For several GPCRs, biased ligands showed better therapeutic value as compared to unbiased ligands as they selectively stimulate desirable receptor-responses while omitting adverse effects regulated by the same receptor [20]. Recently, the first biased GPCR drug Oliceridine (Olinvyk^TM^; aka TRV130) developed by biopharmaceutical company Trevena (Chesterbrook, PA, USA) was approved by the FDA as G protein-biased μ-opioid receptor agonist for the treatment of acute pain that displayed less β-arrestin-mediated adverse effects (e.g., respiratory suppression and nausea) in comparison to morphine [21].

In this study, we employed BRET-based biosensors to measure H_1_R-mediated G_q_ signaling, protein-protein interactions with GRK2, GRK3, GRK5, GRK6, β-arrestin1, and β-arrestin2, and receptor internalization in the same cellular background. Using these BRET-based biosensors, we next aimed to explore whether 2-phenylhistamines (phHA) and histaprodifens (HP) engage different H_1_R responses as compared to histamine (HA) to detect potential biased signaling.

## 2. Results and Discussion

### 2.1. BRET-Based Detection of G_q_ Activation and Signaling by H_1_R

**Figure 1:**
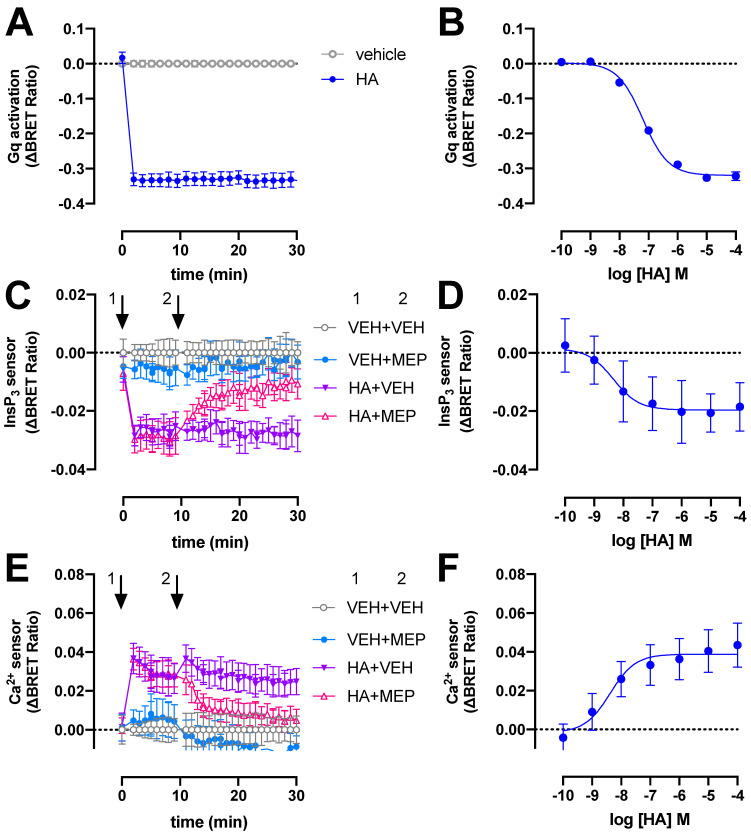
H_1_R activates heterotrimeric G_q_ proteins, InsP_3_ production and Ca^2+^ release in response to histamine (HA). BRET measurements in HEK293T cells transiently co-expressing H_1_R (0.5–1 µg plasmid/dish) in combination with the heterotrimeric G_q_ activation sensor (2.5 µg plasmid/dish) (**A**,**B**), InsP_3_ (**C**,**D**) or Ca^2+^ (**E**,**F**) sensor (4.0–4.5 µg plasmid/dish) in real time upon stimulation with 10 µM histamine (**A**,**C**,**E**) or after 20-min stimulation with increasing concentrations histamine (**B**,**D**,**F**). InsP_3_ and Ca^2+^ cells were first stimulated with vehicle (VEH) or histamine (HA) at t = 0 min followed by a second injection of vehicle (VEH) or 10 µM mepyramine (MEP) at t = 10 min, indicated by the numbered arrows 1 and 2, respectively (**C**–**E**). Data are shown as mean ± SD from at least three independent experiments performed in triplicate.

The H_1_R signals predominantly via heterotrimeric G_q/11_ proteins to stimulate phospholipase C activity, resulting in the production of InsP_3_ and a subsequent increase of intracellular Ca^2+^ levels [2]. To monitor these signaling events in real time we used (1) a recently developed tricistronic BRET-based G_q_ activation sensor plasmid that measures the dissociation of Gβ_3_Gγ_9_-cpVenus heterodimer from Gα_q_-Nanoluc (Nluc) luciferase upon receptor activation [22]; and (2) intramolecular BRET-based InsP_3_ and Ca^2+^ conformation biosensors consisting of the InsP_3_-binding domain of the human type-I InsP_3_ receptor with a R^265^K mutation and a modified Cameleon D3 sensor containing the MLCK calmodulin binding peptide 13 and the D3 variant of calmodulin, respectively [23]. Stimulation of H_1_R with 10 µM histamine (HA) results in rapid activation of heterotrimeric G_q_ proteins reaching a maximum steady-state ΔBRET response within 2 min that remained constant for at least 30 min (Figure 1A). Similar rapid G_q_ activation by H_1_R in response to histamine was previously reported using a fluorescence resonance energy transfer (FRET)-based Gα_q_Gβ_1_Gγ_2_ sensor in HeLa cells [24,25]. In contrast, recruitment of the engineered mini-G_q_ protein to H_1_R was considerably slower as measured using split-NLuc luciferase complementation assay in HEK293T [26], which might be the consequence of the deletion of the membrane anchors and Gβγ-binding interface in its N-terminus [27]. 

The potency (pEC_50_ = 7.2 ± 0.1; mean ± SD; *n* = 4) of histamine to activate heterotrimeric G_q_ proteins in our BRET assay is approximately 4-fold lower than previously observed in a G protein-based transforming growth factor-α shedding assay (Figure 1B) [28]. Surprisingly, a 50-fold lower histamine potency was reported in essentially a similar BRET readout between Gα_q_-Renilla luciferase 8 (Rluc8) and the Gβ_3_Gγ_9_-green fluorescent protein 2 (GFP2) heterodimer (called TRUPATH) in H_1_R expressing HEK293T cells [29], which might be the consequence to different protein expression levels by using a three plasmids-based sensor setup as compared to the single tricistronic biosensor plasmid in the current study [22]. The potency of histamine to induced mini-G_q_ protein to the H_1_R was 10-fold lower as compared our BRET-based G_q_ activation sensor [26], which might be related to the one-to-one protein-protein interaction in the mini-G_q_ assay as opposite to possible activation of multiple heterotrimeric G_q_ proteins by a histamine-bound H_1_R in our BRET-based activation assay (i.e., signal amplification leading to receptor reserve).

Stimulation of H_1_R-expressing HEK293T cells with 10 µM histamine also rapidly increased InsP_3_ and Ca^2+^ levels to steady-state levels within 2 min as measured by ΔBRET changes of the InsP_3_ and Ca^2+^ biosensors, respectively, and this could be reversed by 10 µM H_1_R-antagonist mepyramine (Figure 1C,E). Similar agonist-induced time traces were observed for both these BRET-based InsP_3_ and Ca^2+^ biosensors in HEK293T cells expressing the angiotensin II receptor type 1 or muscarinic acetylcholine receptor M_3_ [23].

Histamine displayed a 10- and 16-fold higher potency to induce the InsP_3_ (pEC_50_ = 8.2 ± 0.8; mean ± SD; *n* = 4) and Ca^2+^ (pEC_50_ = 8.4 ± 0.2; mean ± SD; *n* = 4) response (Figure 1D,F), respectively, as compared to heterotrimeric G_q_ protein BRET sensor activation.

### 2.2. BRET-Based Detection of β-Arrestin1/2 Recruitment to H_1_R

GPCR signaling through heterotrimeric G proteins is generally modulated by the recruitment of β-arrestin1 and/or β-arrestin2 upon phosphorylation of the intracellular GPCR C-tail, resulting in steric hindrance of G protein coupling and subsequent internalization of the GPCR-β-arrestin complex by scaffolding clathrin and the AP2 adaptor [4,5].

**Figure 2:**
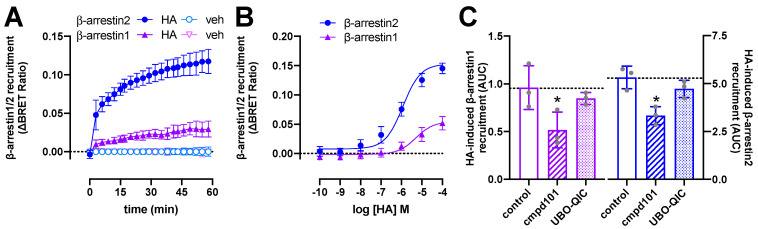
Histamine (HA)-induced β-arrestin1/2 recruitment to H_1_R. BRET measurements in HEK293T cells transiently co-expressing H_1_R-Rluc8 (0.5 µg plasmid/dish) in combination with β-arrestin1-eYFP or β-arrestin2-mVenus (4.0 µg plasmid/dish) in real time upon stimulation with 10 µM histamine (**A**) or after 1-h stimulation with increasing concentrations histamine (**B**). Data are shown as mean ± SD from at least four independent experiments performed in triplicate. Area under the curve (AUC) of 1-h BRET traces in response to 10 µM histamine following 30-min pretreatment of the cells with vehicle (control), 3 µM cmpd101, or 1 µM UBO-QIC. AUC of the BRET experiments are presented as mean ± SD from three independent experiments performed in triplicate, with scatter plots (gray dots) showing the individual AUC values (**C**). Statistical differences (*p* < 0.05) compared to vehicle-pretreated (i.e., control) cells were determined using one-way ANOVA with Dunnett’s multiple comparison test and are indicated by an asterisk.

In frame fusion of the optimized Renilla luciferase 8 (Rluc8) to the H_1_R C-terminal tail did not affect the binding affinity for radiolabeled [^3^H]mepyramine as compared to wildtype H_1_R (Supplementary Figure S1). Stimulation of HEK293T cells with histamine (10 µM) resulted in a gradual increase in ΔBRET between H_1_R-Rluc8 and both β-arrestin1-enhanced yellow fluorescent protein (eYFP) and β-arrestin2-mVenus to reach a steady-state level in approximately 1 h (Figure 2A), as previously observed for β-arrestin2 recruitment to this receptor [6,7]. Histamine induced βarrestin2 recruitment to the H_1_R with comparable potency (pEC_50_ = 6.0 ± 0.3; mean ± SD; *n* = 4) as previously observed in BRET- and split-NLuc-based methods [6,9], whereas a slightly lower potency (pEC_50_ = 5.5 ± 0.5; mean ± SD; *n* = 4) was observed for β-arrestin1 recruitment (Figure 2B). In contrast, 41- and 55-fold higher potencies were observed in a click-beetle split-luciferase setup between H_1_R and β-arrestin1 and 2, respectively [30].

Pretreatment with the Gα_q/11_ protein inhibitor UBO-QIC (1 µM) [31] for 30 min did not significantly affect β-arrestin1/2 recruitment in response to 1-h stimulation with 10 µM histamine (Figure 2C), while completely abolishing histamine-induced nuclear factor activated T-cells (NFAT)-driven reporter gene activation in HEK293T cells expressing H_1_R-Rluc8 (Supplementary Figure S2). In contrast, 30-min pretreatment with the GRK2/3 inhibitor cmpd101 (3 µM) [32] resulted in a partial (~38–46%) but significant reduction in histamine-induced β-arrestin1/2 recruitment (Figure 2C), suggesting that GRK2/3-mediated phosphorylation of H_1_R might in part contribute to the interaction with β-arrestins, as previously observed for several other GPCRs [33,34,35,36,37,38,39,40]. Indeed, overexpression of GRK2 was reported to increase (180%) histamine-induced phosphorylation of H_1_R in HEK293 cells that were metabolically labeled with [^32^P]orthophosphate, whereas GRK5 and GRK6 increased H_1_R phosphorylation by 50 and 80%, respectively [11].

### 2.3. BRET-Based Detection of GRKs Interaction with H_1_R

**Figure 3:**
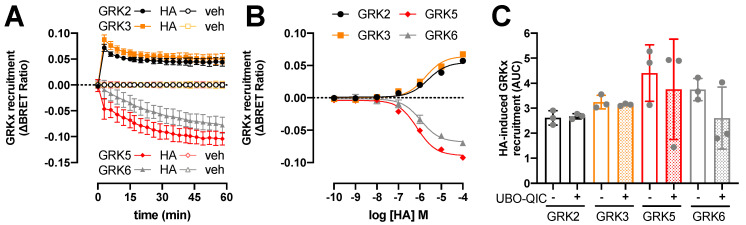
Histamine (HA)-induced modulation in H_1_R interaction with GRKs. BRET measurements in HEK293T cells transiently co-expressing H_1_R-Rluc8 (0.5 µg plasmid/dish) in combination with GRK2-mVenus, GRK3-mVenus, GRK5-mVenus, or GRK6-mVenus (4.0 µg plasmid/dish) in real time upon stimulation with 10 µM histamine (**A**) or after 1-h stimulation with increasing concentrations histamine (**B**). Data are shown as mean ± SD from at least three independent experiments performed in triplicate. Area under the curve (AUC) of 1-h BRET traces in response to 10 µM histamine following 30-min pretreatment of the cells with vehicle (control) or 1 µM UBO-QIC. AUC of the BRET experiments are presented as mean ± SD from three independent experiments performed in triplicate, with scatter plots (gray dots) showing the individual AUC values (**C**). No statistical differences (*p* < 0.05) compared to vehicle-pretreated (i.e., control) cells are observed using one-way ANOVA with Dunnett’s multiple comparison test.

To evaluate which ubiquitously expressed GRK subtypes might potentially phosphorylate the histamine-activated H_1_R and consequently promote β-arrestin1/2 coupling, a BRET assay was used to measure the interaction of H_1_R with GRK2, GRK3, GRK5, and GRK6, as previously reported for the histamine H_4_ receptor (H_4_R) and the atypical chemokine receptor 3 (ACKR3) [33,39]. Histamine (10 µM) induced a very rapid increase in BRET between H_1_R-Rluc8 and GRK2-mVenus or GRK3-mVenus that peaked within 2 min and subsequently decreased within 20 min to an elevated steady-state level as compared to vehicle-stimulated cells (Figure 3A). Hence, GRK2/3 interaction with activated H_1_R indeed precedes the recruitment of β-arrestin1/2 in time, which was also previously reported for agonist-stimulated H_4_R, atypical chemokine receptor 4 (ACKR4) oxytocin, and µ-opioid receptor [33,34,41,42], whereas GRK2/3 and β-arrestin1/2 were recruited with comparable kinetics to ACKR3 in response to chemokine CXCL12 stimulation [39]. On the contrary, histamine (10 µM) stimulation decreased BRET between H_1_R-Rluc8 and GRK5-mVenus or GRK6-mVenus with slower kinetics as compared to the increased GRK2/3 interaction to this receptor, but comparable rates as the recruitment of β-arrestin1/2 (Figure 3A). The histamine-induced BRET decrease suggests that GRK5 and GRK6 are initially in proximity with H_1_R and dissociate upon receptor activation, but the observed response may also reflect a change in conformational orientation between BRET donor and acceptor. In line, reduced BRET between agonist-activated receptor and GRK5 and/or GRK6 has been previously observed for H_4_R, β_2_-adrenergic receptor, bile acid receptor TGR5, neurokinin-1 receptor, and protease-activated receptor 2 [33,43,44,45,46]. In contrast, stimulation of ACKR3 with CXCL12 increased the BRET between this receptor and GRK5 [39]. No agonist-induced changes in BRET signal were observed for GRK6 with ACKR3 nor with bile acid receptor TGR5, and neither for GRK5/6 with ACKR4 [39,42,45].

The potencies of histamine to change the interaction between H_1_R and GRKs 2, 3, 5, and 6 (pEC_50_: 5.6 ± 0.1, 5.7 ± 0.1, 6.1 ± 0.1, and 6.0 ± 0.0, respectively; mean ± SD; *n* = 3) were comparable to its potencies to recruit β-arrestin1/2. Pretreatment of the HEK293T cells with G_q_-inhibitor UBO-QIC (1 µM) for 30 min did not significantly affect the histamine-induced H_1_R interaction with GRK2, 3, 5, and 6 (Figure 3C), indicating that these interactions are independent of heterotrimeric G protein activation as previously shown for H_4_R, β_2_-adrenergic receptor, and dopamine D_2_ receptor [33,36,47].

### 2.4. H_1_R Internalization Requires GRK2/3 and β-Arrestin1/2

**Figure 4:**
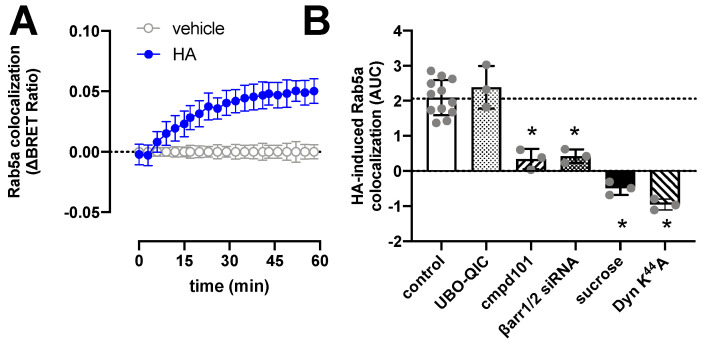
Histamine (HA)-induced internalization of H_1_R. BRET measurements in HEK293T cells transiently co-expressing H_1_R-Rluc8 (0.5 µg plasmid/dish) in combination with the early endosome marker Venus-Rab5a (2 µg plasmid/dish) in real time upon stimulation with 10 µM histamine (**A**). Data are shown as mean ± SD from at least three independent experiments performed in triplicate. Area under the curve (AUC) of 1-h BRET traces in response to 10 µM histamine following 30-min pretreatment of the cells with vehicle (control), 1 µM UBO-QIC, 3 µM cmpd101, or 0.16 µM sucrose, or co-transfection with dominant negative dynamin mutant K^44^A or β-arrestin1/2 siRNA. AUC of the BRET experiments are presented as mean ± SD from three independent experiments performed in triplicate, with scatter plots (gray dots) showing the individual AUC values (**B**). Statistical differences (*p* < 0.05) compared to vehicle-pretreated (i.e., control) cells were determined using one-way ANOVA with Dunnett’s multiple comparison test and are indicated by an asterisk.

H_1_R is internalized upon stimulation with histamine with comparable kinetics as the recruitment of β-arrestin1/2, as measured by the increased BRET between H_1_R-Rluc8 and the early endosome marker Venus-Rab5a (Figure 4A). Pretreatment of HEK293T cells with UBO-QIC, cmpd101, or siRNAs that decrease β-arrestin1/2 expression by 60% (Supplementary Figure S3), revealed that internalization of H_1_R is independent of G_q_ activation but requires GRK2/3 and β-arrestins (Figure 4B). Co-expression of a dominant-negative dynamin K^44^A mutant or hypertonic conditions using 0.16 µM sucrose completely abolished histamine-induced H_1_R translocation towards the early endosomes, indicating that H_1_R internalization is clathrin-dependent as previously reported in transfected Chinese hamster ovary (CHO) cells [48].

### 2.5. Histamine H_1_R Agonists Display Distinct Efficacies in BRET-Based Responses in HEK293T Cells

**Figure 5:**
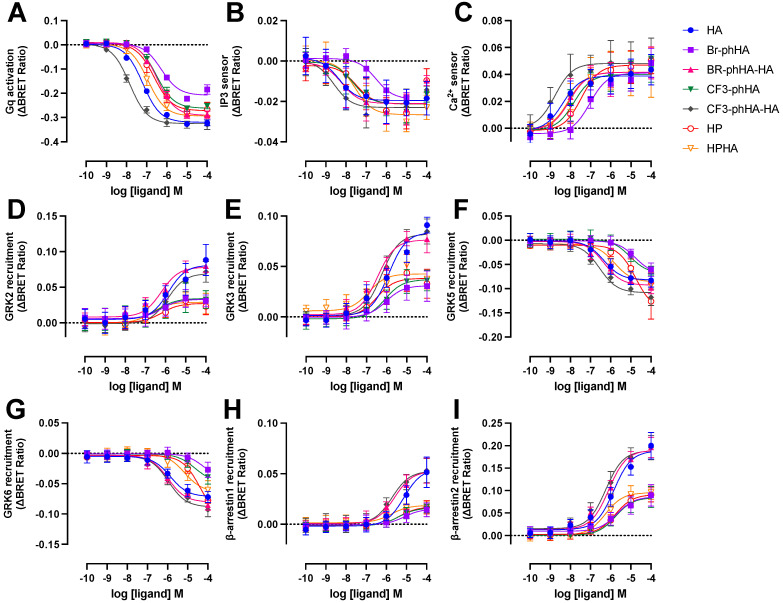
Concentration response curves of BRET-based responses in HEK293T cells upon stimulation with various H_1_R agonists. BRET measurements in HEK293T cells transiently co-expressing H_1_R (0.5–1.0 µg plasmid/dish) in combination with G_q_ activation sensor (2.5 µg plasmid/dish) (**A**), InsP_3_ (**B**) or Ca^2+^ (**C**) sensor (4.0 µg plasmid/dish), or H_1_R-Rluc8 (1.0 µg plasmid/dish) in combination with GRK2-mVenus (**D**), GRK3-mVenus (**E**), GRK5-mVenus (**F**), GRK6-mVenus (**G**), β-arrestin1-eYFP (**H**), or β-arrestin2-mVenus (**I**) (4.0 µg plasmid/dish), upon stimulation with increasing concentrations agonist for 20 (**A**–**C**)or 60 min (**D**–**I**). Data are shown as mean ± SD from at least three independent experiments performed in triplicate.

**Figure 6:**
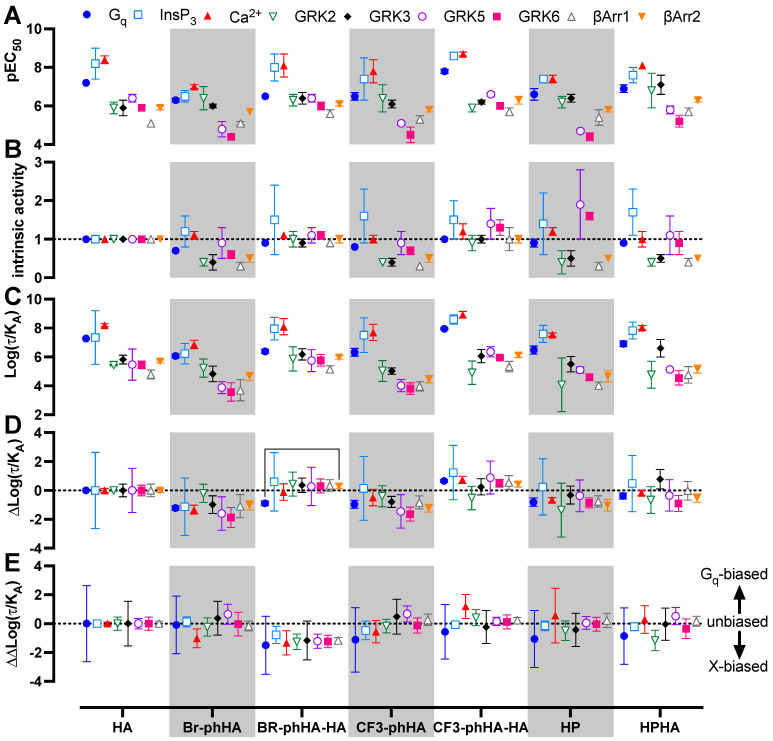
Biased activities of agonists in BRET-based H_1_R responses in transfected HEK293T cells. The pEC_50_ (**A**) and intrinsic activity (**B**) values obtained from the concentration response curves in Figure 5 (Supplementary Tables S1 and S2). Intrinsic activities were calculated versus reference full agonist histamine. Transduction ratio (Log(τ/K_A_) (**C**), relative effectiveness (ΔLog(τ/K_A_) (**D**) and Log bias factor (ΔΔLog(τ/KA) (**E**) as calculated after analyzing the concentration response curves in Figure 5 using the operational model of agonism to retrieve the transduction ratio (Log(τ/K_A_; Supplementary Table S3), followed by normalization to the reference full agonist histamine (ΔLog(τ/K_A_; Supplementary Table S4) and comparison of the various functional readouts to the G_q_ activation response (ΔΔLog(τ/K_A_; Supplementary Table S5). Data are shown as mean ± SD from at least three independent experiments performed in triplicate. Statistical differences (*p* < 0.05) between relative effectiveness of each ligand in the tested responses were determined using Welch ANOVA with Dunnett’s T3 multiple comparison test and are indicated by an asterisk.

The aforementioned BRET-based biosensors were then used to measure H_1_R activation by a selection of agonists (Supplementary Figure S4). Similar to reference agonist histamine, all tested agonists displayed higher potencies to activate G_q_ and in particular the increase in InsP_3_/Ca^2+^ levels, as compared to modulating H_1_R protein-protein interactions with GRK2, GRK3, GRK5, GRK6, β-arrestin1, and β-arrestin2 (Figure 5 and Figure 6A; Supplementary Table S1). Indeed, signal amplification has been previously reported for H_1_R-mediated G protein signaling but was not observed for one-to-one protein-protein interactions such as β-arrestin2 coupling [6]. The tested H_1_R agonists display comparable potencies (0.99- to 3.2-fold difference in EC_50_ values) between InsP_3_ and Ca^2+^ responses, whereas 5- to 37-fold lower potencies were observed for the activation of heterotrimeric G_q_ protein biosensor. However, Br-phHA had only a 1.7- and 5.4-fold lower potency to activate G_q_ as compared InsP_3_ and Ca^2+^ signaling, respectively (Figure 6A; Supplementary Table S1). Interestingly, the difference in potency between GRK2/3 recruitment and G_q_ activation was smaller for Br-phHA, Br-phHA-HA, CF3-phHA, HP, and HPHA (0.6- to 2.5-fold) as compared to histamine (18- and 21-fold, respectively) and CF3-phHA-HA (85- and 36-fold, respectively) (Figure 6A; Supplementary Table S1).

All tested H_1_R agonists acted as full agonists in InsP_3_ and Ca^2+^ signaling, whereas nearly full agonism (intrinsic activities relative to reference agonist histamine α > 0.9) was observed in G_q_ activation for Br-phHA-HA, CF3-phHA-HA, HP, and HPHA (Figure 5A–C and Figure 6B; Supplementary Table S2). The higher intrinsic activities in InsP_3_/Ca^2+^ signaling as compared to G_q_ activation corroborate with the observed increase in potency downstream in the signaling pathway. Comparably high intrinsic activities were previously reported for HP (α = 0.8) and HPHA (α = 0.9) in H_1_R-mediated NF-κB-driven reporter gene activity in COS7 cells [49], for HP (α = 0.96) in inducing G_q_-phospholipase C-β3 interaction in HEK293T cells [50], and for Br-phHA-HA (α = 0.94) and CF3-phHA-HA (α = 0.93) in a steady-state GTPase activity in Spodoptera frugiperda (SF)9 cell membranes co-expressing human H_1_R and RGS4 [51]. In contrast, lower intrinsic activities were previously reporter for HP (α = 0.62) and HPHA (α = 0.64) in this steady-state GTPase activity assay [52], and for HP (α = 0.33) in recruitment of mini-G_q_ protein to H_1_R in a luciferase-complementation assay to measure protein-protein interaction [26].

The intrinsic activities of Br-phHA and CF3-phHA to activate G_q_ activation were 0.7 ± 0.0 and 0.8 ± 0.0 (mean ± SD), respectively (Figure 6B; Supplementary Table S2), which were slightly higher than the α values that were previously observed in the steady-state GTPase activity assay (0.62 and 0.61, respectively) or CRE-driven reporter gene activity (0.56 and 0.62, respectively) [8,51]. As with the reference agonist histamine, both Br-phHA-HA and CF3-phHA-HA acted as full agonists in all used H_1_R assays, whereas Br-phHA, CF3-phHA, HP, and HPHA displayed clear partial agonism in GRK2/3 and β-arrestin1/2 recruitment (Figure 5 and Figure 6B; Supplementary Table S2). In line, partial agonism was previously reported for Br-phHA, CF3-phHA, and HP in a luciferase-complementation-based assay to measure β-arrestin1/2 recruitment to H_1_R [8]. Interestingly, Br-phHA, CF3-phHA, HP, and HPHA might display a higher intrinsic activity to modulate the interaction of H_1_R with GRK5/6 as compared to GRK2/3 and β-arrestin1/2 (Figure 5 and Figure 6B; Supplementary Table S2), although the low potencies of Br-phHA, CF3-phHA, and HP in GRK5/6 BRET assay did not allow accurate determination of their maximum responses.

The presented data in Figure 6A,B suggest signaling bias by some of the H_1_R agonists. For example, relative to reference agonist histamine, full agonist CF3-phHA-HA displays slightly higher potencies (2.4- to 4.0-fold) towards G_q_, InsP_3_, Ca^2+^, and β-arrestin1/2, as compared to the GRK2/3/5/6 responses (1.0- to 2.0-fold higher), suggesting signaling bias towards the G_q_-dependent responses. Oppositely, full agonist Br-phHA-HA shows slightly higher (1.7- to 3.2-fold higher than histamine) normalized potencies for GRK2/3 and β-arrestin1/2 in comparison to G_q_-mediated responses (1.8- to 5-fold lower than histamine), suggesting signaling bias towards GRK2/3 and β-arrestin1/2. Interestingly, HPHA acted as a nearly full agonist in G_q_-mediated responses with 2- to 5-fold lower normalized potencies as compared to histamine, but a partial agonist in the interaction with GRK2/3 and β-arrestins with 2.6- to 14-fold higher normalized potencies than histamine. 

To quantitatively compare the efficacy and potential signaling bias of these H_1_R agonists, all concentration-response data were analyzed using the operational model to retrieve a transduction ratio (τ/K_A_) for each ligand in each response (Figure 6C; Supplementary Table S3) [53,54]. The log (τ/K_A_) values for all agonists were first normalized to the log(τ/K_A_) values of reference agonist histamine within each response to yield Δlog(τ/KA) values as measure of relative effectiveness (Figure 6D; Supplementary Table S4). Both Br-phHA-HA and CF3-phHA-HA displayed slightly higher relative effectiveness in most responses as compared to reference agonist histamine. However, histamine was more effective in G_q_ and GRK2 than Br-phHA-HA and CF3-phHA-HA, respectively. In contrast, Br-phHA, CF3-phHA, and HP displayed slightly lower relative effectiveness than histamine in most responses. Although Br-phHA-HA displayed lower relative effectiveness to induce heterotrimeric G_q_ biosensor activation versus all other tested responses, only a significant bias towards β-arrestin2 recruitment was observed. The relative effectiveness of CF3-phHA-HA to induced GRK2 recruitment to H_1_R was slightly lower as compared to all other tested responses, however, this difference found not to be statistically different. Hence, all H_1_R agonist-induced responses were compared to G_q_ activation by calculating the ΔΔlog (τ/KA) values to quantify bias (Figure 6E; Supplementary Table S5) [53,54,55].

### 2.6. Histamine H_1_R Agonists Induced Ca^2+^ Mobilization in HeLa Cells Endogenously Expressing H_1_R

**Figure 7:**
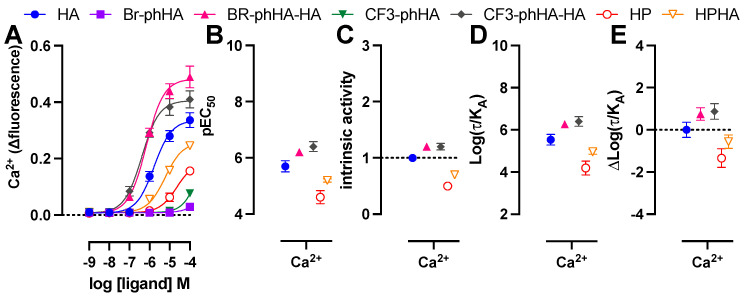
Activities of agonists in endogenous H_1_R-mediated Ca^2+^ response in HeLa cells. Concentration response curves of Ca^2+^ mobilization measured 20–30 s after stimulation of HeLa cells with agonists (**A**). The pEC_50_ (**B**), intrinsic activity (**C**), transduction ratio (Log(τ/K_A_) (**D**), and relative effectiveness (ΔLog(τ/K_A_) as compared to reference agonist histamine (**E**) of agonist-induced Ca^2+^ mobilization in HeLa cells are shown as mean ± SD from at least three independent experiments performed in triplicate.

Finally, HeLa cells were stimulated with the H_1_R agonists to measure their efficacy in the rapid and transient Ca^2+^ mobilization via endogenous H_1_R using the Ca^2+^-sensitive dye Fluo4NW (Figure 7A) [56]. Endogenous expression of H_1_R in HeLa cells (B_max_ = 150–200 fmol/mg protein [24,56]) is approximately 20–34-fold lower as compared to HEK293T cells transfected with 1 µg H_1_R or H_1_R-Rluc8 plasmid (B_max_ = 3000–5000 fmol/mg protein; data not shown). Not surprisingly, all ligands displayed less efficacy for Ca^2+^ mobilization in HeLa cells as compared to G_q_-activation responses in transfected HEK293T as revealed by their considerably lower pEC_50_ values (>30-fold) for most ligands resulting in the (almost) absence of responsiveness to 100 µM Br-phHA and CF3-phHA, while responses by HP and HPHA did not allow accurate determination of their intrinsic activities (Figure 7B,C; Supplementary Tables S1 and S2). However, these lower efficacies in this fast and short term (within 20–30 s) Ca^2+^ response in HeLa cells as opposed to higher efficacies in the more prolonged responses (20–60 min) of the BRET-based assays in HEK293T cells might also be (in part) the consequence of binding kinetics of these agonists to the H_1_R, as observed for dopamine D2 receptor agonists [57]. Both CF3-phHA-HA and Br-phHA-HA displayed higher potency and intrinsic activities as compared to reference full agonist histamine (Figure 7B,C; Supplementary Table S2). In contrast to all other H_1_R agonists, Br-phHA-HA displayed only a 2-fold lower potency to induce Ca^2+^ mobilization in HeLa cells as compared to G_q_-activation in HEK293T cells (Supplementary Table S1). Similar to the BRET-based assays in HEK293T cells, CF3-phHA-HA and Br-phHA-HA displayed higher transduction ratios (log(τ/K_A_) values) and relative effectiveness (Δlog(τ/KA) values) in Ca^2+^ signaling in HeLa cells in comparison to histamine, whereas transduction ratios and relative effectiveness values of HP and HPHA were lower (Figure 7D,E and Supplementary Table S4).

In conclusion, BRET-based assays were used to measure G_q_ activation, InsP_3_ and Ca^2+^ signaling, internalization, and interaction with GRKs2/3/5/6 and β-arrestins1/2 by the H_1_R in response agonist stimulation. Small differences in agonist efficacy were observed between some of these H_1_R responses. However, analysis of these functional responses using the operational model only revealed significant bias of a single agonist, BR-phHA-HA, towards β-arrestin2 recruitment over G_q_ biosensor activation. Nevertheless, these BRET-based biosensors are valuable tools in the pharmacological characterization of H_1_R ligands that can be used in both real-time and end-point format at a reasonably high throughput.

## 3. Materials and Methods

### 3.1. Materials

Fetal bovine serum (FBS) was bought from Bodinco (Alkmaar, The Netherlands) and penicillin/streptomycin was obtained from GE Healthcare (Uppsala, Sweden). Dulbecco’s modified Eagles medium (DMEM, #41966-029), Dulbecco’s phosphate-buffered saline (DPBS, #D8662), trypsin-EDTA, Hanks’ balanced salt solution (HBSS, #14025-050), Pierce^TM^ bicinchoninic acid (BCA) protein assay kit, On-target plus SMARTpool siRNA (β-arrestin1, β-arrestin2, and control), GeneJET gel extraction and GeneJET plasmid miniprep kits, Lipofectamine 2000 were purchased from Thermo Fisher Scientific (Waltham, MA, USA). Poly-L-lysine, histamine, pertussis toxin (PTx), sucrose, Whatman^®^ Westran^®^ PVDF membranes, cOmplete^TM^ protease inhibitor cocktail were purchased from Sigma-Aldrich (St. Louis, MO, USA). UBO-QIC (also known as FR900359) was purchased from the Institute for Pharmaceutical Biology, University of Bonn (Bonn, Germany). Cmpd101 was purchased from Tocris Bioscience (Bristol, UK). Transfection reagent 25 kDa linear polyethylenimine (L-PEI) was bought from Polysciences (Warrington, PA, USA). D-luciferin, coelenterazine-h (CTZ-h), and NanoGlo^®^ were purchased from Promega (Madison, WI, USA). Anti-β-arrestin1/2 clone D24H9 (#4674) and anti-STAT3 clone 79D7 (#4904) were purchased from Cell Signaling Technology (Danvers, MA, USA). Horseradish peroxidase (HRP)-conjugated anti-rabbit secondary antibody was bought from Bio-Rad Laboratories (Hercules, CA, USA). [^3^H]mepyramine (specific activity 20.0 Ci/mmol), MicroScint-O scintillation liquid, and GF/C filter plates were purchased from PerkinElmer (Waltham, MA, USA). Histaprodifen (HP), histaprodifen-histamine dimer (HPHA; Nα-(imidazolylethyl)histaprodifen), 2-(3-bromophenyl)histamine (Br-phHA), 2-(3-bromophenyl)histamine-histamine dimer (Br-phHA-HA), 2-(3-trifluoromethylphenyl)histamine (CF3-phHA), 2-(3-trifluoromethylphenyl)histamine-histamine dimer (CF3-phHA-HA) were a gift from late prof. dr. Walter Schunack (Freie Universität Berlin, Berlin, Germany) and also resynthesized at AstraZeneca (Macclesfield, UK), as previously described [58,59,60]. All other chemicals were of analytical grade and purchased from standard commercial suppliers. Cell culture plastics were purchased from Greiner Bio-One GmbH (Frickenhausen, Germany).

### 3.2. DNA Constructs

Human histamine H_1_ receptor (H_1_R, GenBank: NM_00861), N-terminally hemagglutinin (HA)-tagged H_1_R, H_1_R-Rluc in pcDEF3 expression plasmid have been previously described [6]. Human histamine H_4_ receptor(H_4_R)-Rluc8, β-arrestin1-eYFP, β-arrestin2-mVenus, GRK2-mVenus, GRK3-mVenus, GRK5-mVenus, and GRK6-mVenus in pcDEF3 have been previously described [33]. H_1_R-Rluc8/pcDEF3 was constructed by substituting the Rluc sequence in H_1_R-Rluc with the Rluc8 sequence from H_4_R-Rluc8 using *Not*I and *Xba*I restriction enzymes in the linker and 3’-end flanking sequences, respectively, and verified by DNA sequencing. Dominant-negative dynamin K^44^A and Venus-Rab5a plasmids were kindly provided by Dr. C van Koppen (Essen, Germany) and Dr. N. Lambert (Augusta, GA, USA), respectively [61,62]. The Ca^2+^ and inositol-1,4,5-triphoshate (InsP_3_) bioluminescence resonance energy transfer (BRET) sensors, Luc-D3-M13-cp173-Venus and Luc-IP3R-LBD-R265K-cp173-Venus [23], respectively, were a kind gift from Dr. P. Varnai (Budapest, Hungary). Tricistronic BRET-based G_q_ protein activation biosensors Gβ_3_-T2A-cpVenus-Gγ9-IRES-Gα_q_-Nluc was kindly provided by Dr. H. Schihada (Stockholm, Sweden) [22]. The reporter gene construct pNFAT-luc was obtained from Agilent Technologies (Santa Clara, CA, USA).

### 3.3. Cell Culture and Transfection

Human embryonic kidney 293T (HEK293T) cells (ATCC; Manassas, VA, USA) were maintained in DMEM supplemented with 10% FBS, 50 IU mL^−1^ penicillin and 50 mg mL^−1^ streptomycin in a humidified incubator at 37 °C with 5% CO_2_. HEK293T cells (2 × 10^6^ cells/dish) were seeded in a 10 cm dish and transiently transfected the next day using the PEI method, as previously described [63]. To this end, plasmid DNA was mixed with 20 µg L-PEI (25 kDa) in 0.25 mL NaCl solution (150 mM) and incubated for 30 min at 22 °C before adding dropwise to a 10 cm dish with HEK293T cells. Total DNA amounts were kept at 5 µg by adding empty pcDEF3 plasmid. In β-arrestin knockdown experiments, HEK293T cells (4 × 10^5^ cells/well) were seeded in a 6 well plate and transiently transfected the next day with plasmid DNA (2.5 µg) in combination with 50 nM β-arrestin1 and 50 nM β-arrestin2 siRNAs (1:1) or 100 nM scrambled siRNA using Lipofectamine 2000, as previously described [33]. HeLa cells were previously described [56].

### 3.4. Radioligand Binding Experiments

HEK293T cells were collected two days after transfection with 5 µg HA-H_1_R or H_1_R-Rluc8 plasmids per 10 cm dish in ice-cold phosphate-buffered saline (137 mM NaCl, 2.7 mM KCl, 10 mM Na_2_HPO_4_ and 2 mM KH_2_PO_4_), centrifuged at 1900× *g* for 10 min at 4 °C, and pellets were resuspended and homogenized on ice in binding buffer (50 mM Na_2_HPO_4_ and 50 mM KH_2_PO_4_, pH7.4) using a Branson sonifier 250 (Boom bv, Meppel, The Netherlands). The Protein levels in the cell homogenates was determined using the Pierce^TM^ BCA protein assay kit. Cell homogenates (0.5–1.6 µg/well) were incubated with 0.5 to 80 nM [^3^H]mepyramine for 4 h at 22 °C with gentle agitation in the absence or presence of 10 µM mianserin to quantify total and nonspecific binding, respectively, as previously described [6]. Incubations were terminated by rapid filtration and 3 subsequent washes with ice-cold 50 mM Tris-HCl (pH 7.4) over 0.5% branched polyethyleneimine (750 kDa)-soaked GF/C filter plates using a 96 well FilterMate-harvester (PerkinElmer; Waltham, MA, USA). GF/C filter plates were dried at 52 °C for at least 30 min before addition of 25 µL/well MicroScint-O to quantify filter-bound radioactivity using a Wallac 1450 MicroBeta Trilux counter (PerkinElmer; Waltham, MA, USA) after a delay of at least 3 h. Radioligand binding affinity (K_d_) and total receptor numbers (B_max_) were determined by fitting the data to the “One-site–Total and nonspecific binding” model in GraphPad Prism version 8.4.3 (GraphPad Software, San Diego, CA, USA).

### 3.5. Nuclear Factor Activated T-cells (NFAT)-Driven Reporter Gene Assay

HEK293T cells were collected and seeded in poly-L-lysine-coated white 96-well plates (5 × 10^4^ cells/well) one day after transfection with 1 µg HA-H_1_R in combination with 2.5 µg NFAT-luciferase reporter gene plasmids per 10 cm dish, as previously described with minor modifications [6]. The next day, cells were pre-incubated with 1 µM UBO-QIC or vehicle in serum-free DMEM for 30 min at 37 °C before stimulation with increasing concentrations histamine. The incubations were terminated after 6 h by replacing the medium with 25 µL luciferase assay reagent (0.83 mM ATP, 0.83 mM D-luciferin, 18.7 mM MgCl2, 0.78 µM Na2HPO4, 38.9 mM Tris-HCl (pH 7.8), 0.39% (*v*/*v*) glycerol, 0.03% (*v*/*v*) Triton X-100, and 2.6 µM dithiothreitol) and incubation for 30 min at 37 °C. Luminescence was measured (0.5 s/well) in Mithras LB940 multimode microplate reader (Berthold Technologies, Bad Wildbad, Germany).

### 3.6. Bioluminescence Resonance Energy Transfer (BRET) Assays

HEK293T cells were transiently co-transfected with 0.5–1 µg H_1_R-Rluc8 and 4 µg β-arrestin1-eYFP, β-arrestin2-mVenus, GRK2-mVenus, GRK3-mVenus, GRK5-mVenus, GRK6-mVenus, or 2 µg Venus-Rab5a plasmids per 10 cm dish. For the G protein-activation assay, HEK293T cells were transiently transfected with 0.5 µg H_1_R in combination with 2.5 µg Gβ_3_-T2A-cpVenus-Gγ9-IRES-Gα_q_-Nluc plasmids per 10 cm dish. To detect intracellular levels of InsP_3_ and Ca^2+^, HEK293T cells were transiently transfected with 0.5–1 µg H_1_R in combination with 4.0–4.5 µg Luc-D3-M13-cp173-Venus or Luc-IP3R-LBD-R265K-cp173-Venus plasmids per 10 cm dish. The next day, cells were collected and seeded in poly-L-lysine-coated white 96-well plates (5 × 10^4^ cells/well). Two days after transfection, basal BRET was measured between H_1_R-Rluc8 and the various eYFP/mVenus-fusion proteins using 5 µM coelenterazine-h in HBSS at 37 °C in the Mithras LB940 multimode microplate reader at 540–40 nm (acceptor) and 480–20 nm (donor) or the PHERAstar FS microplate reader (BMG Labtech; Ortenberg, Germany) at 535–30 nm (acceptor) and 475–30 nm (donor) followed by stimulation with H_1_R ligands, as previously described [33]. The G protein-activation sensor was measured in the Clariostar microplate reader (BMG Labtech; Ortenberg, Germany) at 535–30 nm monochromator (acceptor) and 470–80 nm monochromator (donor) using 3.2 μL/mL Nanoglo, as previously described [22,64]. The BRET ratio was first calculated for each well by dividing acceptor by donor light emission, and ligand-induced BRET ratio changes (ΔBRET) were subsequently calculated as ΔBRET = (BRET ratio_ligand_ − BRET ratio_vehicle_)/BRET ratio_vehicle_ [7].

### 3.7. Western Blot

Western blot analysis to detect β-arrestin1/2 expression was performed as previously described [33]. Briefly, HEK293T cells were lysed in RIPA buffer (i.e., 1% NP-40, 0.5% sodium deoxycholate, and 0.1% SDS in PBS) supplemented with 1 mM NaF, 1 mM phenylmethylsulfonyl fluoride, 1 mM Na_3_VO4 and 1x cOmplete^TM^ protease inhibitor cocktail for 20 min on ice, two days after transfection. Next, the lysates were sonicated for 5 s and centrifuged at 20,800× *g* for 10 min at 4 °C. The samples were boiled at 95 °C for 5 min before SDS-PAGE electrophoresis using 10% resolving gels. Next, the proteins were transferred to Whatman^®^ Westran^®^ PVDF membranes and blocked for 1 h in 5% non-fat milk in 0.1% Tween-20/TBS solution at 22 °C. Protein expression was detected using primary antibodies anti-β-arrestin1/2 clone D24H9 (1:1000) and anti-STAT3 clone 79D7, followed by horseradish peroxidase-conjugated secondary antibody (1:5000).

### 3.8. Intracellular Ca^2+^ Mobilization in HeLa Cells

HeLa cells endogenously expressing the H_1_R were seeded in a clear-bottom 96-well plate (2 × 10^4^ cells/well). The next day, the cells were loaded with Fluo-4NW dye (1 vial/24 mL) for one hour in assay buffer (HBSS supplemented with 20 mM HEPES and 2.5 mM probenecid), subsequently washed twice to remove the excess of dye, and reconstituted in assay buffer. Fluorescence (excitation at 494 nm and emission at 516 nm) was measured every second in the NOVOstar microplate reader (BMG Labtech; Ortenberg, Germany) at 37 °C. First the background signal was measured (F_b_), followed by the peak Ca^2+^ mobilization response 5–10 s after agonist injection (F_ago_), finally Triton X-100 (0.25% *v*/*v*) was injected 40 s later to quantify the maximum calcium levels by lysing the cells (F_t_). The agonist-induced Ca^2+^ response was calculated as Δfluorescence = (F_ago_ − F_b_)/(F_t_ − F_b_) [56].

### 3.9. Data Analysis

All results were analyzed using GraphPad Prism v8.4.3 (GraphPad Software, San Diego, CA, USA). Concentration-response curves were fitted using the “three parameters- log(agonist) vs. response” model:

(1) $$response=bottom+\frac{top-bottom}{1+10^{\left(\log\;EC_{50}-\log\left[A\right]\right)}}$$

Intrinsic activity (α) value is calculated as:

(2) $$\alpha =\frac{fitted\;maximum\;response\;agonist}{fited\;maximum\;response\;histamine}$$

Concentration-response curves were globally fitted using the operational model to determine the transduction coefficient log*R* (*R* = τ*/K_A_*) as previously described [53,54,55], with τ and *K_A_* being the index of efficacy and functional equilibrium dissociation constant of the agonist, respectively:

(3) $$response=basal+\frac{\left(E_{max}\;-\;basal\right)R^{n}{\left[A\right]}^{n}}{{\left[A\right]}^{n}R^{n}+{\left(1+\frac{\left[A\right]}{K_{A}}\right)}^{n}}$$

The log*R* value of reference agonist histamine is subtracted from the log*R* values of ligands for each specific response to obtain their relative effectiveness by eliminating the impact of cell- and assay-dependent effects [53,54,55]:

(4) $$\Delta \log R=\log R_{ligand}\;-\;\log R_{histamine}$$

Standard deviation on the Δlog*R* is calculated using:

(5) $$SD_{\Delta \log R}=\sqrt{{\left(SD_{ligand}\right)}^{2}+{\left(SD_{histamine}\right)}^{2}}$$

Next, the Δlog*R* values of each ligand in the different functional responses were subtracted from its Δlog*R* value in G_q_ activation yielding ΔΔlog*R* values to quantify signaling bias between G_q_ activation and the other responses (x). ΔΔlog*R* values > 0 indicate bias towards G_q_ activation over the other responses (x) and vice versa for ΔΔlog*R* values < 0 [53,54,55]:

(6) $$\Delta \Delta \log R=\Delta \log R_{G_{q}\;activation}-\Delta \log R_{response_{x}}$$

Standard deviation on the ΔΔlog*R* is calculated using:

(7) $$SD_{\Delta \Delta \log R}=\sqrt{{\left(SD_{\Delta \log R_{G_{q}\;activation}}\right)}^{2}+{\left(SD_{\Delta \log R_{response_{x}}}\right)}^{2}}$$

Welch ANOVA (does not assume equal variance) with a Dunnett’s T3 multiple comparisons test on the Δlog*R* values was used to determine the statistical significance of ligand activity between pathways, where *p* < 0.05 was considered to be significant.

## Data Availability

Data is contained within the article or Supplementary Materials.

## Supplementary Materials

The following are available online at https://www.mdpi.com/article/10.3390/ijms23063184/s1.
