# Supplementary material for: BRET-Based Biosensors to Measure Agonist Efficacies in Histamine H1 Receptor-Mediated G Protein Activation, Signaling and Interactions with GRKs and β-Arrestins
Source: Int J Mol Sci. 2022 Mar 16;23(6):3184. doi: 10.3390/ijms23063184 (PMC8953162; doi:10.3390/ijms23063184)
Supplement: Supplementary file 1 [file ijms-23-03184-s001.zip › ijms-1627962-supplementary.pdf]

International Journal of
Molecular Sciences

MDPI

Supplementary materials

BRET-Based Biosensors to Measure Agonist Efficacies in Histamine H1 Receptor-Mediated G Protein Activation, Signaling and Interactions with GRKs and β-Arrestins

Eléonore W. E. Verweij †, Reggie Bosma †, Meichun Gao, Jelle van den Bor, Betty Al Araaj, Sabrina M. de Munnik, Xiaoyuan Ma, Rob Leurs, and Henry F. Vischer *

Division of Medicinal Chemistry, Faculty of Science, Amsterdam Institute of Molecular and Life Sciences, Vrije Universiteit Amsterdam, 1081 HZ Amsterdam, The Netherlands; noortje.verweij@xs4all.nl (E.W.E.V.); r.bosma@vu.nl (R.B.); m.c.gao@vu.nl (M.G.); j.vanden.bor@vu.nl (J.v.d.B.); bettyalaraaj@hotmail.com (B.A.A.); sabrinademunnik@hotmail.com (S.M.d.M.); x.ma@vu.nl (X.M.); r.leurs@vu.nl (R.L.)

* Correspondence: h.f.vischer@vu.nl

† These authors contributed equally to this work.

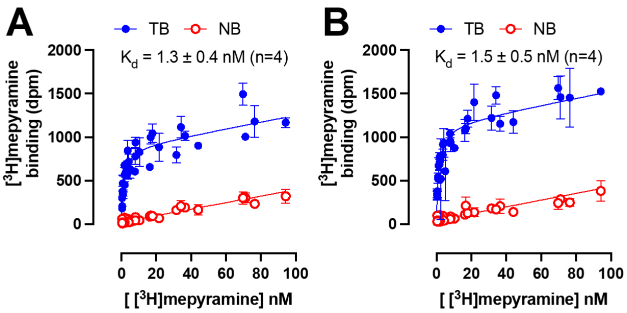

Figure S1. Saturation binding of [^{3}H]mepyramine to H_1R and H_1R-Rluc8. Total (TB) and non-specific (NB) binding of [^{3}H]mepyramine to homogenates prepared from HEK293T cells expressing either H_1R (**A**) or H_1R-Rluc8 (**B**). Non-specific binding was determined in the presence of 10 µM mianserin. Pooled data are shown as mean ± SD from 4 independent experiments performed in triplicate.

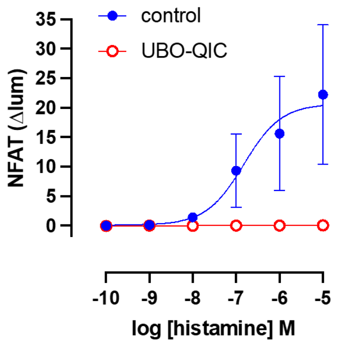

Figure S2. Inhibition of H_1R signaling to NFAT reporter gene by G_q inhibitor UBO-QIC. NFAT-driven reporter gene activity was measured in HEK293T cells co-transfected with H_1R in response to increasing histamine concentrations in the absence or presence of 1 µM UBO-QIC. Data are shown as mean ± SD from 3 independent experiments performed in triplicate.

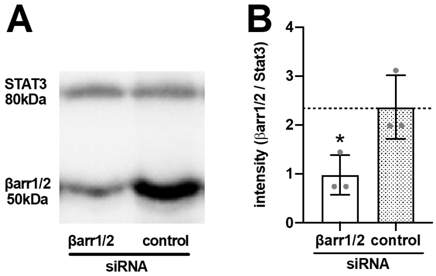

Figure S3. Depletion of β-arrestin1/2 proteins by siRNA. Representative β-arrestin1/2 immunoblot of HEK293T cells co-expressing H_1R-Rluc8 and Venus-Rab5a upon treatment with scrambled or β-arrestin1/2 siRNA (**A**). STAT3 expression was used as loading control. Densitometric quantification (mean ± SD; scatter plot of individual data) from 3 independent experiments using ImageJ software (National Institutes of Health, MD, USA) (**B**). Statistical difference ($p < 0.05$) compared to scrambled siRNA-treated cells was determined with unpaired t-test and indicated by an asterisk.

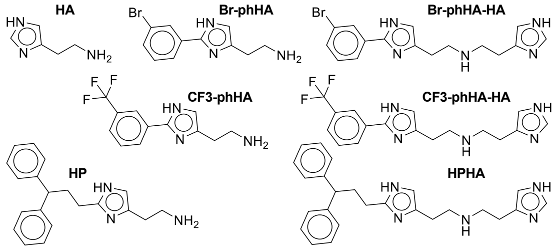

Figure S4. Chemical structures of H_1R agonists used in this study: histamine (HA), 2-(3-bromophenyl)histamine (Br-phHA), 2-(3-bromophenyl)histamine-histamine dimer (Br-phHA-HA), 2-(3-trifluoromethylphenyl)histamine (CF3-phHA), 2-(3-trifluoromethylphenyl)histamine-histamine dimer (CF3-phHA-HA), Histaprodifen (HP), and histaprodifen-histamine dimer (HPHA; Nα-(imidazolylethyl)histaprodifen).

Table S1. Potency (pEC_{50}) values determined for H_1R agonists in different functional responses

	G_q	$InsP_3$	Ca^{2+}	GRK2	GRK3	GRK5	GRK6	βArr1	βArr2	Ca^{2+} (HeLa)
HA	7.2±0.1 (4)	8.2±0.8 (4)	8.4±0.2 (4)	5.9±0.3 (3)	5.9±0.4 (3)	6.4±0.2 (3)	5.9±0.0 (3)	5.1±0.0 (3)	5.9±0.1 (4)	5.7±0.4 (4)
Br-phHA	6.3±0.1 (3)	6.5±0.3 (3)	7.0±0.1 (3)	6.4±0.6 (3)	6.0±0.1 (3)	4.8±0.4 (3)	4.4±0.0 (3)	5.1±0.1 (3)	5.7±0.0 (4)	ND (3)
Br-phHA-HA	6.5±0.0 (3)	8.0±0.7 (3)	8.1±0.6 (3)	6.3±0.3 (3)	6.4±0.3 (3)	6.4±0.2 (3)	6.0±0.2 (3)	5.6±0.2 (3)	6.1±0.1 (4)	6.2±0.1 (3)
CF3-phHA	6.5±0.2 (3)	7.4±1.1 (3)	7.8±0.6 (3)	6.4±0.7 (3)	6.1±0.2 (3)	5.1±0.1 (3)	4.5±0.4 (3)	5.3±0.2 (3)	5.8±0.1 (4)	ND (3)
CF3-phHA-HA	7.8±0.1 (3)	8.6±0.1 (3)	8.7±0.1 (3)	5.9±0.2 (3)	6.2±0.1 (3)	6.6±0.1 (3)	6.0±0.1 (3)	5.7±0.2 (3)	6.3±0.2 (4)	6.4±0.3 (3)
HP	6.6±0.3 (3)	7.4±0.0 (3)	7.4±0.2 (3)	6.2±0.3 (3)	6.4±0.2 (3)	4.7±0.1 (3)	4.4±0.2 (3)	5.4±0.4 (3)	5.8±0.1 (4)	4.6±0.4 (3)
HPHA	6.9±0.2 (3)	7.6±0.4 (3)	8.1±0.0 (3)	6.8±0.9 (3)	7.1±0.5 (3)	5.8±0.2 (3)	5.2±0.3 (3)	5.7±0.2 (3)	6.3±0.1 (4)	5.2±0.1 (3)

Potency (pEC_{50}) values were determined for H_1R agonists after 20-minutes stimulation of HEK293T cells co-expressing H_1R (1.0 µg plasmid/dish) with heterotrimeric G_q activation, $InsP_3$, or Ca^{2+} BRET sensors, after 60-minutes stimulation of HEK293T cells co-expressing H_1R-Rluc8 (1.0 µg plasmid/dish) in combination with GRK2-mVenus, GRK3-mVenus, GRK5-mVenus, GRK6-mVenus, β-arrestin1-eYFP, or β-arrestin2-mVenus (Figure 5), or after 5-10-seconds stimulation of Fluo4-NW-loaded HeLa cells that endogenously express H_1R (Figure 7A). Data are shown as mean ± standard deviation with number of independently performed experiments indicated between parentheses. ND = no agonist response detected.

Table S2. Intrinsic activity (α) values determined for H_1R agonists in different functional responses.

	G_q	$InsP_3$	Ca^{2+}	GRK2	GRK3	GRK5	GRK6	βArr1	βArr2	Ca^{2+} (HeLa)
HA	1.0±0.0 (4)	1.0±0.0 (4)	1.0±0.0 (4)	1.0±0.0 (3)	1.0±0.0 (3)	1.0±0.0 (3)	1.0±0.0 (3)	1.0±0.0 (3)	1.0±0.0 (4)	1.0±0.0 (4)
Br-phHA	0.7±0.0 (3)	1.2±0.4 (3)	1.1±0.1 (3)	0.4±0.1 (3)	0.4±0.2 (3)	0.9±0.4§ (3)	0.6±0.1§ (3)	0.3±0.1 (3)	0.5±0.1 (4)	ND (3)
Br-phHA-HA	0.9±0.0 (3)	1.5±0.9 (3)	1.1±0.0 (3)	1.0±0.2 (3)	0.9±0.1 (3)	1.1±0.2 (3)	1.1±0.1 (3)	0.9±0.0 (3)	1.0±0.1 (4)	1.2±0.1 (3)
CF3-phHA	0.8±0.0 (3)	1.6±0.7 (3)	1.0±0.1 (3)	0.4±0.0 (3)	0.4±0.1 (3)	0.9±0.3§ (3)	0.7±0.1§ (3)	0.3±0.0 (3)	0.5±0.1 (4)	ND (3)
CF3-phHA-HA	1.0±0.0 (3)	1.5±0.5 (3)	1.2±0.2 (3)	0.9±0.2 (3)	1.0±0.1 (3)	1.4±0.4 (3)	1.3±0.2 (3)	1.0±0.3 (3)	1.0±0.1 (4)	1.2±0.1 (3)
HP	0.9±0.1 (3)	1.4±0.8 (3)	1.2±0.1 (3)	0.4±0.3 (3)	0.5±0.2 (3)	1.9±0.9§ (3)	1.6±0.1§ (3)	0.3±0.1 (3)	0.5±0.0 (4)	0.5±0.0 (3)
HPHA	0.9±0.0 (3)	1.7±0.6 (3)	1.0±0.2 (3)	0.4±0.1 (3)	0.5±0.1 (3)	1.1±0.5 (3)	0.9±0.3 (3)	0.4±0.1 (3)	0.5±0.0 (4)	0.7±0.0 (3)

Intrinsic activity (α) values were calculated by dividing the fitted maximum responses of H_1R agonist by reference full agonist histamine. Fitted maximum responses were determined for H_1R agonists after 20-minutes stimulation of HEK293T cells co-expressing H_1R (1.0 µg plasmid/dish) with heterotrimeric G_q activation, $InsP_3$, or Ca^{2+} BRET sensors, after 60-minutes stimulation of HEK293T cells co-expressing H_1R-Rluc8 (1.0 µg plasmid/dish) in combination with GRK2-mVenus, GRK3-mVenus, GRK5-mVenus, GRK6-mVenus, β-arrestin1-eYFP, or β-arrestin2-mVenus (Figure 5), or after 5-10-seconds stimulation of Fluo4-NW-loaded HeLa cells that endogenously express H_1R (Figure 7A). Data are shown as mean ± standard deviation with number of independently performed experiments indicated between parentheses. [§]potency too low for accurate Emax determination. ND = no agonist response detected.

Table S3. Log(τ/K_A) values determined for H_1R agonists in different functional responses.

	**G_q**	**$InsP_3$**	**Ca^{2+}**	**GRK2**	**GRK3**	**GRK5**	**GRK6**	**βArr1**	**βArr2**
HA	7.28±0.07 (3)	7.35±1.86 (3)	8.20±0.11 (3)	5.43±0.04 (3)	5.82±0.31 (3)	5.47±1.08 (3)	5.45±0.26 (3)	4.79±0.31 (3)	5.69±0.10 (4)
Br-phHA	6.07±0.14 (3)	6.23±0.70 (3)	6.86±0.29 (3)	5.23±0.63 (3)	4.83±0.54 (3)	3.88±0.41 (3)	3.58±0.41 (3)	3.69±0.73 (3)	4.65±0.29 (4)
Br-phHA-HA	6.39±0.14 (3)	7.96±0.78 (3)	8.09±0.56 (3)	5.87±0.83 (3)	6.18±0.39 (3)	5.75±0.76 (3)	5.77±0.40 (3)	5.14±0.26 (3)	5.97±0.12 (4)
CF3-phHA	6.32±0.27 (3)	7.51±1.20 (3)	7.70±0.56 (3)	5.03±0.73 (3)	5.02±0.22 (3)	4.02±0.42 (3)	3.81±0.40 (3)	3.96±0.32 (3)	4.46±0.25 (4)
CF3-phHA-HA	7.95±0.06 (3)	8.59±0.31 (3)	8.93±0.22 (3)	4.91±0.81 (3)	6.06±0.46 (3)	6.36±0.35 (3)	5.96±0.06 (3)	5.33±0.36 (3)	6.11±0.14 (4)
HP	6.48±0.28 (3)	7.60±0.59 (3)	7.54±0.12 (3)	4.07±1.86 (3)	5.50±0.53 (3)	5.10±0.21 (3)	4.59±0.23 (3)	4.02±0.24 (3)	4.66±0.39 (4)
HPHA	6.91±0.20 (3)	7.83±0.58 (3)	8.03±0.14 (3)	4.77±0.93 (3)	6.60±0.61 (3)	5.13±0.09 (3)	4.55±0.50 (3)	4.76±0.57 (3)	5.16±0.27 (4)

Log(τ/K_A) values were determined for H_1R agonists after 20-minutes stimulation of HEK293T cells co-expressing H_1R (1.0 µg plasmid/dish) with heterotrimeric G_q activation, $InsP_3$, or Ca^{2+} BRET sensors, or after 60-minutes stimulation of HEK293T cells co-expressing H_1R-Rluc8 (1.0 µg plasmid/dish) in combination with GRK2-mVenus, GRK3-mVenus, GRK5-mVenus, GRK6-mVenus, β-arrestin1-eYFP, or β-arrestin2-mVenus (Figure 5). Data are shown as mean ± standard deviation with number of independently performed experiments indicated between parentheses. ND = no agonist response detected.

Table S4. ΔLog(τ/K_A) values determined for H_1R agonists in different functional responses.

	**G_q**	**$InsP_3$**	**Ca^{2+}**	**GRK2**	**GRK3**	**GRK5**	**GRK6**	**βArr1**	**βArr2**
HA	0.00±0.10	0.00±2.63	0.00±0.10	0.00±0.05	0.00±0.44	0.00±1.53	0.00±0.36	0.00±0.43	0.00±0.14
Br-phHA	-1.22±0.15	-1.13±1.99	-1.33±0.31	-0.20±0.63	-0.98±0.62	-1.60±1.16	-1.87±0.69	-1.09±0.80	-1.04±0.31
Br-phHA-HA	-0.89±0.16	0.60±2.02	-0.11±0.57	0.44±0.83	0.36±0.50	0.28±1.33	0.31±0.48	0.35±0.40	0.28±0.16
CF3-phHA	-0.96±0.28	0.15±2.21	-0.49±0.57	-0.40±0.73	-0.79±0.38	-1.45±1.16	-1.64±0.48	-0.83±0.45	-1.23±0.27
CF3-phHA-HA	0.66±0.09	1.24±1.88	0.74±0.25	-0.52±0.81	0.25±0.56	0.89±1.14	0.51±0.26	0.55±0.48	0.42±0.17
HP	-0.81±0.29	0.25±1.95	-0.66±0.16	-1.36±1.87	-0.32±0.62	-0.37±1.10	-0.86±0.34	-0.76±0.39	-1.03±0.40
HPHA	-0.38±0.21	0.48±1.95	-0.17±0.18	-0.66±0.93	0.78±0.68	-0.34±1.09	-0.90±0.56	-0.02±0.65	-0.53±0.29

ΔLog(τ/K_A) values were determined for H_1R agonists after 20-minutes stimulation of HEK293T cells co-expressing H_1R (1.0 µg plasmid/dish) with heterotrimeric G_q activation, $InsP_3$, or Ca^{2+} BRET sensors, or after 60-minutes stimulation of HEK293T cells co-expressing H_1R-Rluc8 (1.0 µg plasmid/dish) in combination with GRK2-mVenus, GRK3-mVenus, GRK5-mVenus, GRK6-mVenus, β-arrestin1-eYFP, or β-arrestin2-mVenus (Figure 5). Data are shown as mean ± standard deviation.

Table S5. $\Delta\Delta$Log(τ/K_A) values and bias factors determined for H_1R agonists in different functional responses.

	**G_q-InsP$_3$**	**G_q-Ca^{2+}**	**G_q-GRK2**	**G_q-GRK3**	**G_q-GRK5**	**G_q-GRK6**	**G_q-βArr1**	**G_q-βArr2**
HA	0.00±2.63(1.00)	0.00±0.14(1.00)	0.00±0.11(1.00)	0.00±0.45(1.00)	0.00±1.54(1.00)	0.00±0.37(1.00)	0.00±0.45(1.00)	0.00±0.17(1.00)
Br-phHA	-0.09±1.99(0.81)	0.12±0.34(1.31)	-1.02±0.65(0.10)	-0.23±0.64(0.59)	0.38±1.17(2.39)	0.65±0.70(4.52)	-0.11±0.81(0.93)	-0.17±0.34(0.67)
Br-phHA-HA	-1.50±2.02(0.03)	-0.78±0.60(0.16)	-1.33±0.84(0.05)	-1.26±0.53(0.06)	-1.17±1.34(0.07)	-1.21±0.50(0.06)	-1.24±0.43(0.06)	-1.17*±0.22(0.07)
CF3-phHA	-1.12±2.23(0.07)	-0.47±0.64(0.34)	-0.56±0.78(0.27)	-0.17±0.47(0.68)	0.49±1.20(3.07)	0.68±0.55(4.80)	-0.13±0.53(0.74)	0.27±0.39(1.86)
CF3-phHA-HA	-0.57±1.89(0.26)	-0.07±0.26(0.85)	1.19±0.82(15.37)	0.42±0.56(2.62)	-0.23±1.14(0.59)	0.16±0.28(1.43)	0.12±0.48(1.31)	0.24±0.19(1.75)
HP	-1.06±1.97(0.09)	-0.15±0.33(0.71)	0.56±1.89(3.59)	-0.49±0.68(0.32)	-0.43±1.14(0.37)	0.05±0.45(1.13)	-0.04±0.48(0.91)	0.22±0.49(1.68)
HPHA	-0.86±1.96(0.14)	-0.21±0.27(0.62)	0.29±0.96(1.93)	-1.16±0.71(0.07)	-0.04±1.11(0.92)	0.52±0.60(3.32)	-0.35±0.68(0.45)	0.16±0.35(1.43)

Log(τ/K_A) values were determined for H_1R agonists after 20-minutes stimulation of HEK293T cells co-expressing H_1R (1.0 μg plasmid/dish) with heterotrimeric G_q activation, InsP$_3$, or Ca^{2+} BRET sensors, or after 60-minutes stimulation of HEK293T cells co-expressing H_1R-Rluc8 (1.0 μg plasmid/dish) in combination with GRK2-mVenus, GRK3-mVenus, GRK5-mVenus, GRK6-mVenus, β-arrestin1-eYFP, or β-arrestin2-mVenus (Figure 5). Data are shown as mean ± standard deviation. Bias factors were calculated as $10^{\Delta\Delta Log(\tau/KA)}$ and are shown between parentheses. Statistical differences ($p < 0.05$) between ΔLog(τ/K_A) values of each ligand in the tested responses were determined using Welch ANOVA with Dunnett's T3 multiple comparison test and is indicated with an asterisk.
